# Use of Biomarker Data and Relative Potencies of Mutagenic Metabolites to Support Derivation of Cancer Unit Risk Values for 1,3-Butadiene from Rodent Tumor Data

**DOI:** 10.3390/toxics10070394

**Published:** 2022-07-15

**Authors:** Christopher R. Kirman, Sean M. Hays

**Affiliations:** Summit Toxicology, Bozeman, MT 59715, USA; shays@summittoxicology.com

**Keywords:** biomarkers, internal dose, species differences, mixtures assessment, benchmark dose, cancer unit risks

## Abstract

Unit Risk (UR) values were derived for 1,3-butadiene (BD) based upon its ability to cause tumors in laboratory mice and rats. Metabolism has been established as the significant molecular initiating event of BD’s carcinogenicity. The large quantitative species differences in the metabolism of BD and potency of critical BD epoxide metabolites must be accounted for when rodent toxicity responses are extrapolated to humans. Previously published methods were extended and applied to cancer risk assessments to account for species differences in metabolism, as well as differences in mutagenic potency of BD metabolites within the context of data-derived adjustment factors (DDEFs). This approach made use of biomarker data (hemoglobin adducts) to quantify species differences in the internal doses of BD metabolites experienced in mice, rats, and humans. Using these methods, the dose–response relationships in mice and rats exhibit improved concordance, and result in upper bound UR values ranging from 2.1 × 10^−5^ to 1.2 × 10^−3^ ppm^−1^ for BD. Confidence in these UR values was considered high based on high confidence in the key studies, medium-to-high confidence in the toxicity database, high confidence in the estimates of internal dose, and high confidence in the dose–response modeling.

## 1. Introduction

1,3-Butadiene (BD) is manufactured for producing styrene–butadiene rubber, polymers, and other chemicals. BD is associated with increased leukemia and bladder/urinary cancer mortality in some synthetic rubber workers following exposures to high concentrations [1,2]; it also produces tumors at multiple sites in laboratory rodents, for which mice are demonstrably more sensitive than rats [3,4,5]. In past cancer risk assessments for BD, mouse tumors have served as the primary basis for rodent-based estimates of cancer potency (Table 1). 

Metabolism of BD to multiple reactive metabolites plays an important role in its carcinogenic mode of action (MOA). The weight of evidence for species differences in the metabolic activation of BD is robust (see Section 2.2). Due to large species differences in metabolism, mice, rats, and humans are exposed to vastly different internal doses of reactive metabolites for a given external exposure to BD, which likely underlies large species differences in carcinogenic potency (mice >> rats). Motwani and Tornqvist [10] relied upon metabolite-specific biomarkers (e.g., hemoglobin adducts), second-order rate constants for adduct formation, and species-specific erythrocyte lifespans to quantify species differences in internal doses of BD metabolites (e.g., AUC for metabolites in blood) in mice, rats, and humans. This approach is considered here to reflect the best available science for BD dosimetry, and is applied to the derivation of data-derived extrapolation factor (DDEF) values for BD [11] (USEPA, 2014). The goal of this manuscript is to extend and apply the approach of Motwani and Tornqvist [10] and Fred et al. [12] to support the derivation of rodent-based cancer unit risk (UR) values for BD.

## 2. Background

This section provides background information on previous dose–response assessments for tumors in rodents, metabolism, and mode of action (MOA) studies for BD. These data are used here to support key decisions in the cancer dose–response assessments conducted for BD. In so doing, these data reduce the uncertainty associated with using rodent-derived data for BD human health risk assessment consistent with USEPA guidelines [11,13].

### 2.1. Previous Rodent-Based Cancer Risk Assessments for BD

Previous rodent-based cancer risk assessments conducted by regulatory agencies and risk assessors are summarized in Table 1. These potency estimates take into consideration tumor incidence at multiple tissue sites in mice (Table 2) and rats (Table 3) exposed to BD via inhalation. The rodent carcinogenicity database for BD is robust, and includes acute cancer bioassays in mice of both sexes [14], a series of stop-exposure studies conducted in male mice (NTP, 1993), and lifetime cancer bioassays in mice and rats of both sexes [3,4]. Upper bound cancer potency estimates based on mouse tumor data are significantly higher than corresponding values based on rat tumor data, which is generally attributed to underlying differences between species in metabolic activation and detoxification (see Section 2.2). Notably, nearly all rodent-based risk assessments for BD in the past relied upon a default approach of relying on the concentration of BD in air. Accounting for quantitative differences in BD metabolism between species represents an important challenge for human health risk assessment, including those planned for BD in the U.S. under TSCA regulations [15]. More recent data and methods published since the time of these risk assessments now allow for these important differences to be addressed quantitatively in human health cancer risks assessments for BD (see Section 3).

### 2.2. Metabolism Overview

**Figure 1:**
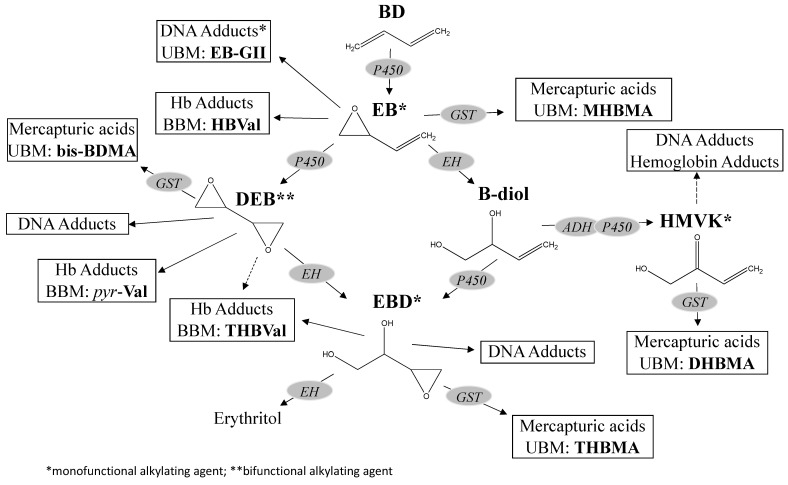
1,3-Butadiene metabolism.

BD is chemically inert, but is metabolized to several electrophilic epoxides that are capable of alkylating cellular macromolecules, to which the genotoxic and carcinogenic effects of BD are attributed (see Section 2.3 below). The metabolism of BD to reactive epoxide metabolites, including 2,3-epoxy-1-butene (EB), 1,2,3,4-diepoxybutane (DEB), and 3,4epoxybutane-1,2-diol (EBD), has been well studied in mice, rats, and humans (as reviewed in Himmelstein et al. [16], Albertini et al. [17], Kirman et al. [18], Filser et al. [19], which indicates that the metabolic pathways for BD are qualitatively similar, but exhibit large quantitative differences across species. Internal doses of these metabolites reflect pathways accounting for their formation (e.g., oxidation) as well as their clearance (e.g., hydrolysis, conjugation) as depicted in Figure 1.

Large species differences in the metabolism of BD are consistently reported in in vitro, in situ, and in vivo studies. In vitro studies on Michaelis–Menten constants (Vmax and Km values) for activation and detoxification pathways of BD in microsomes indicate that mice have a significantly higher ratio of EB activation-to-detoxification than either rats or humans [10,20,21,22,23,24,25]. In the effluent of mouse livers perfused with BD, all three epoxides (EB, DEB, and EBD) and BD-diol were observed, while in effluents from rat livers perfused with BD, only EB and BD-diol were detected. When the mouse and rat livers were perfused with EB, Filser et al. [19,26] found that BD-diol, EBD, and DEB were formed, with BD-diol predominating in both species. DEB formation was greater in mouse than in rat livers [19]. Following in vivo exposures of rats and mice to BD via inhalation, differences in circulating DEB levels have been reported to be over 100-fold greater in mice than in rats [27,28,29].

Quantitative differences in the in vivo production of BD metabolites are also reflected in the accumulation of metabolite-specific hemoglobin adducts. A DEB-specific hemoglobin adduct, N,N-(2,3-dihydroxy-1,4-butadiyl)-valine (pyr-Val), has been identified and measured, providing insights into species and exposure differences in BD metabolism [30]. The formation of pyr-Val hemoglobin adducts has been studied in male and female mice and rats exposed to 1.0 ppm by inhalation for 6 h/day for 4 weeks [31], in which adduct burdens (i.e., concentrations in blood due to cumulative exposure) in rats were more than 30-fold lower than the corresponding values in mice. The formation of pyr-Val adducts in rats and mice of both sexes was assessed following 4-week inhalation exposures to either 1, 6.25, or 62.5 ppm BD for 6 h/day [32]. The difference in adduct levels between species was large (mice > rats by approximately 1 order of magnitude) and dose-dependent, with larger differences observed at higher concentration compared to low concentrations. Swenberg et al. [31] compared results of occupationally exposed workers in the Czech Republic to results of BD-exposed mice and rats for pyr-Val. Pyr-Val adducts were not detected (LOD of 0.3 pmol/g Hb) in occupationally exposed men and women, with the mean exposures ranging from 0.18–0.8 ppm [17,33]. Using analytical methods with improved sensitivity, Swenberg et al. [34] and Boysen et al. [35] detected pyr-Val in humans. For a given exposure to BD, DEB blood levels in humans (estimated from measured pyr-Val adducts) were approximately 16-fold lower than the DEB blood levels in rats, which, in turn, are approximately 45-fold lower than the DEB blood levels in mice.

Motwani and Tornqvist [10] estimated internal doses (i.e., blood AUCs per unit exposure) for BD metabolites in mice, rats, and humans using two approaches: (1) estimating blood dose from hemoglobin adduct data using second-order rate constants for adduct formation and erythrocyte half-lives, and (2) scaling up metabolite clearance rates from in vitro studies. For DEB, both approaches yielded consistent results, in which large differences were estimated across species (mice > rats > humans). Of primary importance to human health risk assessments, relative species differences in DEB AUC between mice and humans are very large (approximately 2 to 3 orders of magnitude) [10]. Based on hemoglobin adduct biomarkers [10] and urinary biomarker data [36], there is clear evidence that mice, rats, and humans are exposed internally to mixtures of BD metabolites that are qualitatively similar, but have important quantitative differences.

### 2.3. Mode of Action Summary

There is clear evidence from in vivo and in vitro studies that BD can produce genotoxicity through the formation of electrophilic metabolites (as reviewed in USEPA [6]; Albertini et al. [37]). USEPA [6] concluded *“...it is virtually certain that the carcinogenic effects are mediated by genotoxic metabolites of 1,3-butadiene.*” Key events for a genotoxic mode of action, which can include both point mutations (e.g., mutagenic MOA) and/or clastogenic events (e.g., clastogenic MOA) were summarized in Kirman et al. [38]: (1) Exposure to BD; (2) Distribution of BD to metabolizing tissues (liver); (3) Metabolism of BD to electrophilic intermediates (epoxide metabolites); (4) Distribution of electrophilic intermediates to target tissues; (5) Formation of DNA adducts; (6) Error in DNA replication; (7) Viable cell with gene mutation; and (8) Tumor Progression. Because metabolic activation (Key event 3) is considered the molecular initiating event in the MOA, quantification of the large species differences in metabolism serves as an important challenge to quantitative risk assessment.

BD, through its metabolism, is both mutagenic and clastogenic. The types of genotoxic events (point mutations vs. chromosomal aberrations) may play differing roles in the various cancers associated with BD exposure in rodents and humans. Point mutations are generally assumed to play an initiating role in the carcinogenic process, and often serve as the basis for assumptions of low-dose linearity as a matter of risk assessment policy. However, specific chromosomal aberrations are known to play a key role in some human leukemias (e.g., Philadelphia chromosome and chronic myelogenous leukemia), but interestingly, were not observed in human cells exposed to DEB in vitro, despite increases in DNA double-strand breaks [39]. For cancer types requiring clastogenic events (e.g., reciprocal translocations/deletions), a nonlinear dose–response relationship may better reflect the underlying mode of action for specific structural chromosome alterations requirement of two hits during a single round of DNA replication for their production [40]. The aberrations will arise at a frequency proportional to the square of the exposure concentration, and therefore, cancers that are dependent upon reciprocal translocations or interstitial deletions are expected to exhibit a quadratic component to their dose–response relationship.

## 3. Methods

### 3.1. Exposure Concentration and Duration (CxT) in Tumor Response

An assessment of the relative importance of exposure concentration and exposure duration was conducted using methods adapted from ten Berge et al. [41]. Specifically, tumor incidence data from the male mice stop-exposure studies and lifetime cancer bioassay data for select tumors types (lymphoma, heart, lung) (Table 2) were used to create log–log plots of predicted exposure concentrations resulting in a 10% increase in tumor response (EC10 values) vs. exposure duration. EC10 values were first estimated for lifetime exposure data in male mice using the multistage model (USEPA BMDS, version 3.2; U.S. Environmental Protection Agency, Washington DC, USA), since these datasets included the most test groups. The form of the multistage model (1st through 5th degree polynomial) was then applied and adjusted to match single data points for responses following less-than-lifetime exposures from the stop-exposure study in male mice. The less-than-lifetime adjusted model was then used to estimate EC10 values for each duration assessed in the stop-exposure study. A linear regression model was fit to the natural log of the EC10 values as a function of exposure duration (natural log of days), and the slope of the regression was used to interpret the relative importance of concentration and duration in the observed tumor response. Specifically, a slope of −1 is expected when concentration and exposure duration are of equal importance to tumor response (i.e., consistent with the default assumption for Haber’s conjecture). Similarly, a slope between −1 and 0 serves to indicate that concentration is of greater importance than exposure duration, and a slope that is less (i.e., more negative) than −1 indicates that exposure duration is of greater importance than concentration.

### 3.2. Unit Risk Derivation

Cancer UR values for BD were calculated from rodent bioassay data in a manner generally consistent with USEPA methodology [13,42] (USEPA, 2005, 2012) using the following equation:

*UR = BMR/POD_HEC_* (1)

where

*UR* = unit risk (per ppm, continuous exposure);

*BMR* = benchmark response rate (e.g., 10%); 

*POD_HEC_* = point of departure (e.g., benchmark dose) expressed in terms human equivalent concentration (ppm, continuous exposure), after adjusting for discontinuous exposures and species differences in the toxicokinetics of BD.

UR derivation is a multistep process that includes key decisions for: (1) human equivalent concentration calculation; (2) endpoint/dataset selection; (3) dose–response modeling; (4) point of departure (POD) selection; (5) low-dose extrapolation; and (6) additional adjustments.

#### 3.2.1. Human Equivalent Concentration Calculation

As discussed in Section 2.2, there are clear species differences in the metabolism of BD that need to be accounted for when calculating human equivalent concentrations (HECs) from test concentrations of BD administered to rodents. Accordingly, HECs were calculated using the following equation:

HEC = (TC * AF)/EF_AK_ (2)

where

*HEC* = human equivalent concentration (ppm, continuous exposure);

*TC* = test concentration administered to mice or rats (ppm, discontinuous exposure);

*AF* = adjustment factor to account for discontinuous exposure in toxicity studies (e.g., 6 h/24 h per day, 5 days/7 days per week); 

*EF_AK_* = data-derived extrapolation factor (DDEF) to account for species differences in the toxicokinetics of BD.

DDEF values were derived in a manner generally consistent with USEPA [11] guidelines (see Supplementary Materials). Based upon consideration of the uncertainty in the mode of action for systemic tumors (see Section 2.3), the reactive metabolites of BD (EB, DEB, EBD) are assumed to each contribute to carcinogenic effects of BD. Although DEB is considered to be the most potent metabolite of BD with respect to genotoxicity (see MOA discussion above), a potential role for other reactive metabolites cannot be ruled out. To estimate the combined contribution of BD metabolites in a quantitative manner (dose additivity assumed), a genotoxicity index approach was applied using the following equation:

(3) $$GI_{S}=\sum [(Unit\;AUC_{EB}\times RP_{EB})+(Unit\;AUC_{DEB}\times RP_{DEB})+(Unit\;AUC_{EBD}\times RP_{EBD})]$$

where

*GI_S_* = species-specific genotoxicity index, calculated separately for male and female mice, rats, and humans (nM*h per ppm*h BD);

*Unit AUC* = species-specific unit AUCs for each metabolite, which reflects the internal dose of each metabolite in each species for a given exposure to BD (nM*h per ppm*h BD; Table 4).

*RP* = relative potency of each metabolite for producing genotoxicity in in vitro cell systems (unitless; summarized in Table 5 and derived in Supplementary Materials).

Accordingly, *EF_AK_* values for interspecies extrapolations can be calculated using a ratio approach [11], as defined by the following equation:

(4) $$EF_{AK}=\frac{C_{A}}{C_{H}}=\frac{GI_{H}}{GI_{A}}$$

where

*C_A_* = air concentration in animals producing an internal dose of cytotoxic equivalents at or near the point of departure, AUC_A_ (ppm);

*C_H_* = air concentration in humans producing an internal dose of cytotoxic at or near the point of departure, AUC_A_ (ppm);

*EF_AK_* = data-derived extrapolation factor for interspecies extrapolation due to toxicokinetic differences (unitless);

*GI* = genotoxicity index for BD metabolites per ppm BD in laboratory animals (A) or humans (H) (ppm^−1^) (Table 6).

#### 3.2.2. Endpoint/Dataset Selection

Target tissues for cancer risk assessment were selected based upon a review of risk assessments by regulatory agencies and risk assessors available for 1,3-butadiene (Table 1), and based on a review of the recently published literature. Datasets used to estimate the cancer potency of BD include the lifetime cancer bioassay incidence data for the following:

Female Mice–lymphoma, histiocytic sarcoma, mammary gland, ovary, Harderian gland, liver, forestomach, lung, heart tumors (Table 2);

Male Mice–lymphoma, histiocytic sarcoma, preputial, kidney, Harderian gland, liver, forestomach, alveolar–bronchiolar, heart tumors (Table 2);

Female Rats–uterus, mammary gland, thyroid, and Zymbal gland tumors (Table 3); 

Male Rats–pancreas, testes, and glial cell tumors (Table 3).

Incidence data for acute exposures (all sexes and species) were not used to estimate cancer potency since no significant tumor incidences were reported. Similarly, incidence data from the stop-exposure study in male mice were not used to estimate cancer potency, but were instead used to the support supplemental CxT analyses (see Section 3.1)

#### 3.2.3. Benchmark Dose Modeling

Each tumor dataset was modeled separately using the multistage model (1st through 5th degree polynomial) (BMDS, version 3.2; U.S. Environmental Protection Agency, Washington DC, USA). The best fitting degree of the multistage model was selected based on a consideration of AIC, goodness of fit *p*-value, and visual inspection.

The multistage model was used to estimate the EC10 value, as well as its 95% lower confidence limit (LEC10), 95% upper confidence limit (UEC10), and the cumulative distribution function (CDF; 1st–99th percentile values). Within each species, a distribution of endpoint-specific unit risk values was determined using the CDFs generated by BMDS and Equation (1) (i.e., 10%/EC10). A distribution for the multisite unit risk value was calculated for each species/sex by summing across cancer endpoints:

(5) $$UR_{Combined}=\sum \left(UR_{Endpoint1}+UR_{Endpoint2}\ldots \right)$$

where

*UR_Combined_* = combined unit risk across endpoint calculated for each sex and species (ppm^−1^); 

*UR_Endpoint_* = tumor endpoint-specific unit risk within each sex/species (ppm^−1^).

A distribution for the combined UR values was generated using Monte Carlo methods (Crystal Ball; Excel; version 7.3; Oracle, Austin, TX, USA) based on a simulation of 10,000 iterations. The 5th and 95th percentiles for the combined UR distributions were adopted as the lower and upper confidence limits, respectively, for each combined dataset.

## 4. Results

### 4.1. Exposure Concentration and Duration (CxT)

**Figure 2:**
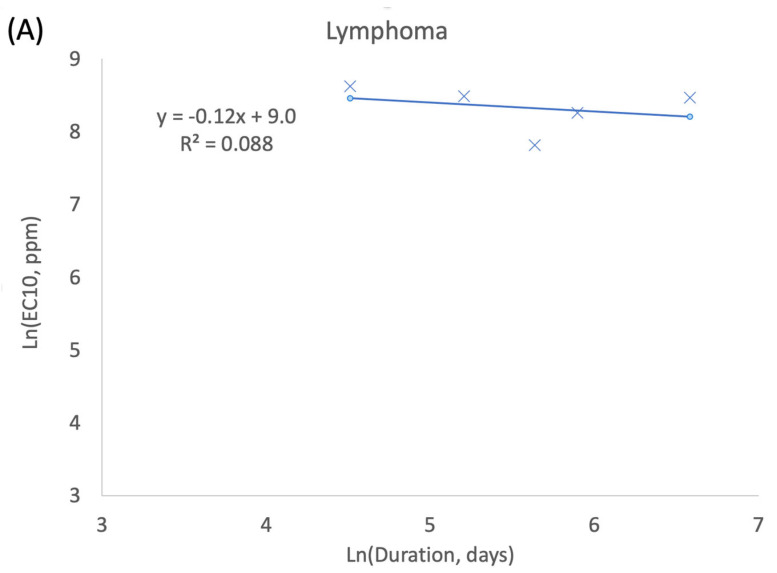

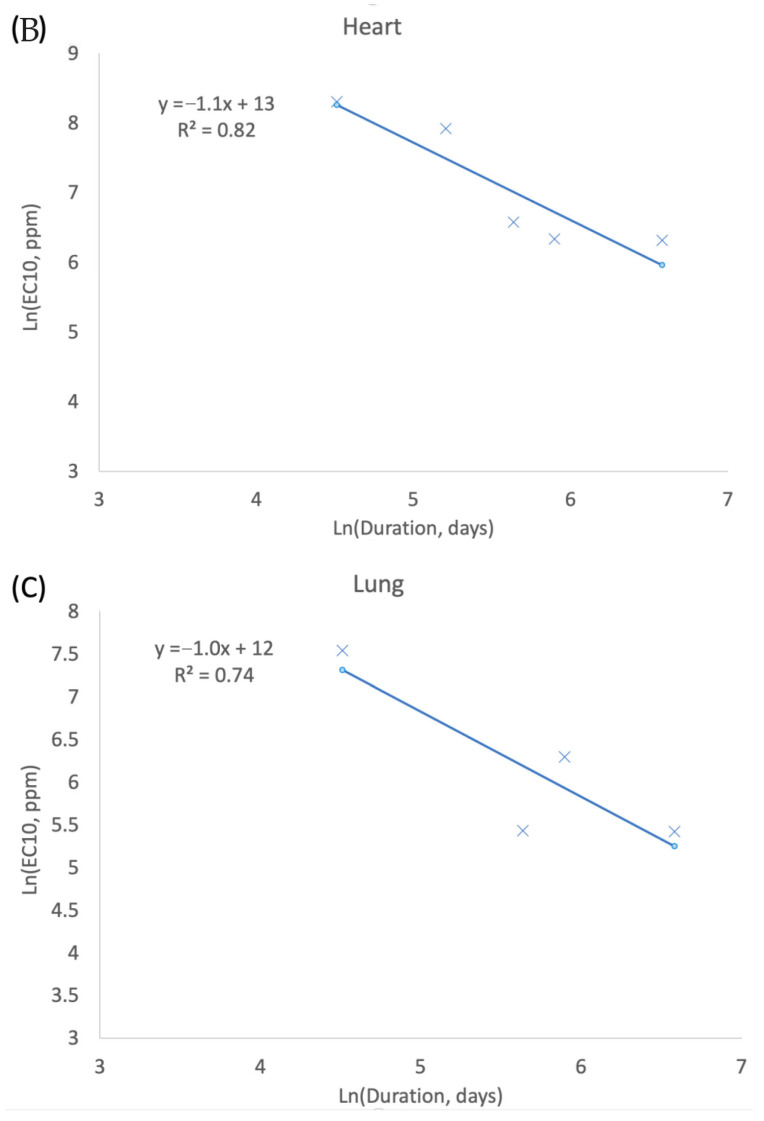
CxT assessment for select tumors in male mice exposed to BD (lifetime and stop-exposure study data): (**A**) lymphomas; (**B**) heart tumors; (**C**) lung tumors.

Log–Log plots of predicted EC10 values vs. exposure duration for select tumors in male mice are provided in Figure 2. For solid tumor type slope terms of the linear regression for heart tumors (−1.1) and lung tumors (−1.0) are both approximately equivalent to the expected value of −1. For these tumor types, exposure concentration and exposure duration are considered to be equally important to the observed tumor response, a result that is consistent with Haber’s conjecture. In contrast, the slope term for lymphomas (−0.12) regression indicates that the concentration term is much more important than the exposure duration term for the observed cancer response. This result is inconsistent with Haber’s conjecture, and suggests that there may be important mechanistic differences in BD’s role in producing mouse lymphomas compared to the solid tumors observed in mice.

### 4.2. Human Equivalent Concentrations

EF_AK_ values of 0.0300, 0.0228, 0.531, and 0.556 were calculated to extrapolate to humans from female mice, male mice, female rats, and male rats, respectively (Table 6). These values account for species differences in the internal dose of reactive epoxide metabolites (Table 4), as well as metabolite differences in genotoxic potency (Table 5). For comparison purposes, EF_AK_ values of 0.000886, 0.000630, 0.0165, and 0.0175 were similarly calculated for DEB alone, for instance, if an alternative the hypothesis were adopted assuming the clastogenic effects of DEB are solely responsible for the observed tumor response (i.e., assuming contributions of EB and EBD tumor response are negligible). The approach for BD used here is similar to that proposed by Fred et al. [12] to address differences in the genotoxic potency of BD metabolites for cancer endpoints for tumors observed in mice and rats, but has been expanded to include humans as well as additional datasets for assessing relative genotoxic potency.

**Figure 3:**
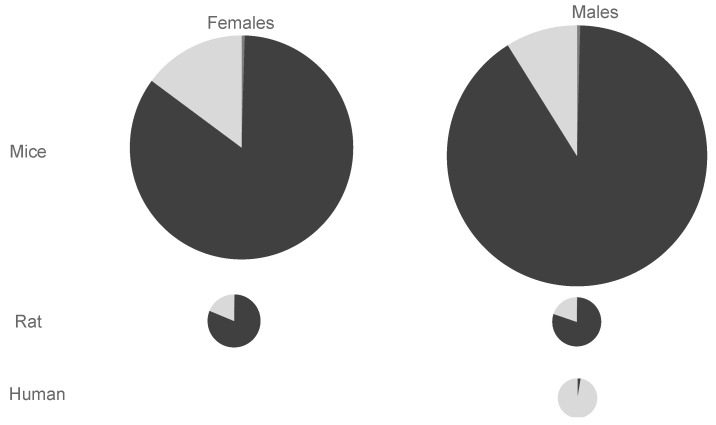
Species differences in the genotoxicity index: pie surface area is proportionate to total cytotoxicity index. Metabolite contributions: DEB = dark shading; EB = medium shading; EBD = light shading.

Based on these DDEF calculations, species differences in the genotoxicity index per unit BD exposure are depicted in Figure 3, which shows that values are highest (largest pie surface area) in mice, followed by rats, and then humans. In rodents, DEB is the largest contributor to the genotoxicity indices, with moderate contributions from EBD, while contributions from EB are negligible. Sex differences in rodents for the genotoxicity indices and relative metabolite contributions are small. In humans, EBD is clearly the largest contributor to the genotoxicity index, and DEB and EB are negligible.

### 4.3. Unit Risk Values and Species Concordance

**Figure 4:**
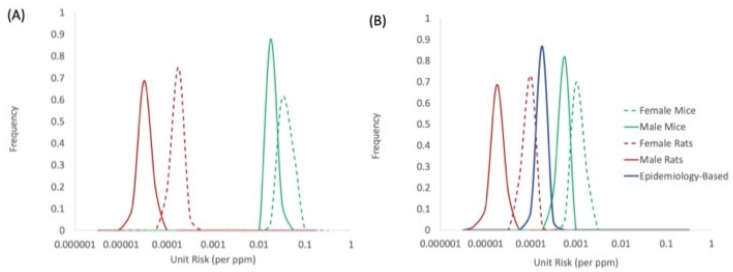
Concordance of unit risk distributions: (**A**) unadjusted exposure and (**B**) adjusted for species differences in internal dose and genotoxic potency of BD metabolites; unit risk values based on epidemiology data are from Valdez-Flores et al. [50].

Central tendency (maximum likelihood estimation, MLE) estimates for unit risk values (90% CIs in parentheses) based on combined target tissue cites were determined to be 0.00088 (0.00057–0.0012), 0.00035 (0.00028–0.00043), 0.000067 (0.000042–0.000096), and 0.000014 (0.0000075–0.000021) (ppm^−1^) based on data for female mice, male mice, female rats, and male rats, respectively (Table 7). A comparison of the species-specific distributions for BD unit risk values is provided in Figure 4. This comparison shows that the data-derived extrapolation factor adjustments to account for species differences in metabolic activation of BD improves the overall concordance across species, as evidenced by the reduced spread of the distributions in the adjusted unit risk values (Figure 4B) compared to unadjusted values (Figure 4A). In addition, the range of adjusted unit risk values based on rodent tumor data compares reasonably well to the unit risk distribution derived from epidemiology data for exposed styrene–butadiene rubber (SBR) workers based on data for leukemia and bladder cancer [50]. This unit risk value is based on a Cox proportional hazard regression for the two cancer endpoints, based on the most recent follow-up and exposure data [1,2].

### 4.4. Consideration of Sensitive Subpopulations and Additional Adjustments

As a matter of policy, the derivation of unit risk values should also consider potentially sensitive subpopulations [13]. Potential sensitivity to the carcinogenic effects of BD can be attributed to toxicokinetic and/or toxicodynamic factors, as summarized below.

With respect to toxicokinetics, the mode of action for BD’s carcinogenic action involves the metabolic activation of reactive epoxides [18,37]. Blood and urinary biomarker data for BD can be used to characterize human variation in metabolism due to: (1) gender differences; (2) ethnicity differences; and (3) genetic polymorphisms. Gender differences have been reported for men and women occupationally exposed to BD with respect to hemoglobin adducts (Vacek et al., 2010) and urinary biomarkers [36]. When expressed on a per mg/m^3^ BD exposure basis, these differences are approximately 2-fold (females < males). Ethnicity differences, generally less than a factor of 2, have been reported for urinary biomarkers for BD metabolites, including significantly higher concentrations of MHBMA in White Americans as compared to Japanese Americans and Native Hawaiians [51], and significantly higher concentrations of DHBMA in African Americans compared to White Americans [52]. In addition, ethnic differences in urinary excretion of repaired DNA adducts (EB-GII) have been reported [53,54]. Differences across ethnic groups are generally up to 2- to 3-fold. Some of the ethnic differences in BD biomarkers may be related to known genetic polymorphisms across ethnic groups [55,56,57,58] (, especially GSTT1 gene copy number [52]. In vitro studies have shown that human cell lines of differing glutathione-S-transferase (GST-T1) status differ in sensitivity to EB (GSTT1- cells exhibiting greater sensitivity than GSTT1 + cells [59]). The effects of genetic polymorphisms for various enzyme systems (P450, GST, EH) alone and combined were assessed for the DEB-specific hemoglobin adduct levels (THBVal). Specific polymorphisms (particularly for GSTT1) showed significant effects on THBVal levels [60]. THBVal levels across different metabolism groups (i.e., combinations of genetic polymorphisms) were found to be generally within a factor of 2 of the overall mean. The weight of evidence from available biomarker studies on BD suggests that human variation based on toxicokinetic (TK) factors is likely near or below the default uncertainty factor for intraspecies variation (i.e., UFtk ≲ 3).

With respect to toxicodynamics, due to the fact that BD is metabolized to reactive epoxides capable of producing genotoxic events, conditions and disease states associated with reduced repair of DNA damage are expected to be potentially sensitive to the carcinogenic effects of BD. For example, sensitivity to BD metabolite and clastogen, 1,2,3,4-diepoxybutane (DEB), is specifically used in the diagnosis of Fanconi’s anemia [61]. However, quantification of potential risks to specific disease states exposed to BD is beyond the scope of this paper.

As a matter of policy, genotoxic chemicals such as BD are expected to pose an increased risk when exposures occur early in life, a time period that is not directly covered by data from animal cancer bioassays or epidemiology studies of occupational cohorts. Some evidence is available for BD that suggests early life exposures are not associated with increased risk. For example, BD is metabolically activated to epoxide metabolites by cytochrome P450, principally isozyme CYP2E1. Based on the ontogenesis of CYP2E1 activity in humans [62,63,64] (, metabolic activation of BD is expected to be much lower in neonates, infants, and children, compared to adults. However, this age trend also holds for the enzyme systems (e.g., glutathione-S-transferase [64]) that detoxify the reactive metabolites of BD, and therefore, the net impact of age on susceptibility is less clear. Biomarker data for BD reflect the net balance between age differences in metabolic activation and detoxification. Based on NHANES biomonitoring data in the U.S., BD biomarkers were nominally higher in children (3–5 years) and adolescents (6–19 years), compared to adults (>20 years) [65]. However, these differences are less than would be expected based on age differences in inhalation rates and body weights [66,67], suggesting that, after adjusting for inhalation/body weight differences, age-dependent metabolism factors contributing BD biomarkers are lower in children compared to those in adults.

Lastly, acute cancer bioassays conducted in the most sensitive species (mice) indicate that single, high exposure to BD (1000, 5000, or 10,000 ppm) relatively early in life does not initiate tumors over the course of their lifetime [14. For these reasons, the application of an ADAF may not be required to ensure the protection of human health from BD exposures.

## 5. Discussion and Conclusions

A series of analyses were conducted using the robust rodent tumor data available for BD to determine concordance for human health risk assessment. From these analyses, several conclusions can be supported:

(1) Risk from Acute Exposures—Acute exposures, even those associated with extremely high levels of BD in the most sensitive species, do not appear to be associated with an increased risk of cancer. An apparent duration threshold, which falls between 1 day (no increase in tumors observed) and 91 days (the shortest duration with an observed increase in tumors) likely exists for BD tumors in mice. For this reason, quantitative cancer risk assessment may not be required for acute human exposures scenarios to BD.

(2) Haber’s Conjecture—For chronic and lifetime exposures to BD, cumulative exposure (e.g., concentration x time, or ppm/days) appears to serve as an appropriate dose measure for some cancer types (e.g., mouse lung, heart). This conclusion is similar to that made using SBR worker cohort data for BD exposure and leukemia and urinary/bladder cancers [50]. In contrast, for mouse lymphomas, the concentration term appears to be of greater importance to response than time (duration). Important mechanistic differences may underly the departure from Haber’s conjecture noted for mouse lymphoma response, and the relevance of this endpoint to human health is questionable given the differing results reported for human leukemia.

(3) Use of DDEF Adjustments for Interspecies Extrapolation—By accounting for species differences in metabolic activation, concordance across species in the cancer potency estimates for BD is improved (Figure 4B). Remaining differences across species in BD cancer potency may reflect toxicodynamic differences, for which no adjustments were made. To reconcile the differences in cancer potencies of BD in mice and rats in Figure 4B (i.e., for the distributions to overlap) would require the existence of a toxicodynamic difference of approximately 13- to 25-fold between the two species (mouse > rat).

UR values were derived for BD based upon tumor incidences reported in laboratory mice and rats following lifetime exposures. In both species, cancer potency estimates were higher in female animals compared to male animals. This gender difference is not supported by hemoglobin adduct data, which show similar or higher production of BD metabolites in male animals (e.g., for DEB formation male mice > female mice). Instead, this gender difference likely reflects the presence of additional target tissues in female animals compared to males (e.g., mammary gland tumors; Table 2 and Table 3). This difference may be limited to laboratory rodents, since breast cancer mortality was not associated with BD exposure in female workers [1,2,50], and an increased potency of BD is not supported by human data. The UR values derived here are considerably lower than those derived previously by regulatory agencies (see Table 1), since they account for species differences in metabolic activation. Substantial species differences in the metabolism of BD result in humans and rodents experiencing internal doses of reactive metabolites that are qualitatively similar (i.e., all three reactive metabolites are formed in all species), but exhibit large quantitative differences. Accounting for these differences serves as an important challenge to human health risk assessment. The methods of Fred et al. [12] and Motwani and Tornqvist [10] were extended and applied to this assessment to account for species differences in metabolism, as well as differences in metabolite mutagenic potency. This approach made use of biomarker data (metabolite-specific hemoglobin adducts) to quantify species differences in the internal doses of BD metabolites experienced in mice, rats, and humans. The use of hemoglobin adducts for BD here is consistent with USEPA’s practice in the assessment of other chemicals (e.g., acrylamide risk assessment [68]). The availability of biomonitoring data across species enables a data-driven approach to better place rodent tumor results into the context of human equivalent exposure.

Although the use of a relative potency approach to address the contributions from mixtures of metabolites originating from a single chemical may be viewed as novel, this approach has been applied to risk assessments for chemical mixtures that are believed to act via a common mechanism, including polycyclic aromatic hydrocarbons [69] and dioxin-like chemicals [70], and are therefore justified here.

Sources of uncertainty are identified for this assessment below.

Mode of Action—For this assessment, it was assumed that all three epoxide metabolites contribute to the tumor responses observed in rodents. Because DEB is a bifunctional genotoxic agent that is capable of producing clastogenic effects, an alternative hypothesis that DEB is solely responsible for the observed tumor responses can be supported. Based on the DDEF values derived for DEB alone compared to those derived for the combined action of all three metabolites (Table 6), UR values based on DEB alone would be approximately 32- to 36-fold lower than derived here, which is consistent with the comparatively low levels of DEB produced by humans. For this reason, the assumption that all three BD epoxide metabolites contribute to cancer risk is considered to be a health protective assumption.

Relative Potency Approach—The relative genotoxic potency estimates calculated here (EB = 1.0, DEB = 85, EDB = 1.5) can be compared to the mutagenic potencies estimated by Fred et al. [12] for these three metabolites (EB = 1.0, DEB = 32, EDB = 0.21). The genotoxic potency of EDB was higher in this assessment, largely based on the results of Meng et al. [45], who reported considerable differences in potency across the four stereoisomers of EBD, for which some stereoisomers (e.g., 2R,3S-EBD) were found to be more potent than EB. More recently, Nakamura et al. [71] reported that all four stereoisomers of EBD were of similar potency, and that the results of Meng et al. [45] may have been due to a contaminant. The approach used in this assessment could be expanded to address stereochemistry differences in metabolism, with the collection of data to characterize species differences in stereoisomer formation and their internal doses. However, some uncertainty remains regarding the potential contribution of other BD metabolites to adverse effects, including hydroxymethylvinyl ketone (HMVK), as well as proposed chlorohydroxy metabolites produced via myeloperoxidase [72,73,74]. The approach used in this assessment could be expanded to include additional BD metabolites if their importance is warranted from a mechanistic standpoint, and if the data needed to estimate internal doses (e.g., from hemoglobin adduct data) and relative potencies are generated.

Human Equivalent Concentration Calculation—Uncertainty in the internal dose estimates calculated from hemoglobin adducts per Motwani and Tornqvist [10] is considered low. Some uncertainty remains in the use of hemoglobin adduct data collected from male workers (Table 4) to estimate internal doses in human populations that include males and females. Small differences (<2-fold) in internal dose estimates are noted between male and female mice, and between male and female rats [10,34]. However, these observations in rodents differ from findings in humans, which show similar or lower formation of adducts in women compared to men [34].

Dose–Response Modeling—Uncertainty in the dose–response modeling is considered to be relatively low. The BMD:BMDL ratios for the combined UR values calculated for each sex and species, which serve as overall indicators of the uncertainty associated with fitted model parameters, were found to be less than a factor of 1.5 (Table 7) in this assessment.

Despite these sources of uncertainty, overall confidence in the UR values derived for BD here is high. The key datasets are defined by well-conducted studies that have been consistently selected by regulatory agencies to support cancer risk assessments for BD. Confidence in the dosimetry of the assessment is also considered high, since they are derived from excellent biomarker data that are metabolite-specific and have been quantified in all three species of interest (mice, rats, and humans). Confidence in the cancer database is considered high, since the carcinogenicity of BD has been well-studied in rodents and humans, and the database is considered robust.

## Figures and Tables

**Table 1:** Summary of rodent-based cancer risk assessments for 1,3-butadiene.

Assessor (Year)	Endpoint	Dataset	DR Model	POD Type	POD Value	Species Extrapolation Assumption	Low-Dose Extrapolation Assumption	Unit Risk (ppm^−1^)
USEPA [6]	Leydig cell, pancreatic exocrine cell, Zymbal gland, mammary gland, thyroid follicular cell	Male and Female Rats [7]	Multistage	LEC10	NS	Air concentration equivalence	Linear	0.0042–0.056
	Lymphocytic lymphomas, histiocytic sarcomas, heart hemangiosarcomas, lung, forestomach, Harderian gland, liver, preputial gland, ovary, mammary gland	Male and Female Mice [3]	Multistage-Weibull time-to-tumor	LEC10	0.7–13.3 ppm	Air concentration equivalence	Linear	0.0064–0.29
Health Canada [8]	Multiple	Male and Female Rats [7]	Multistage	TC05	4.7–905 mg/m^3^	Air concentration equivalence	NA	0.00012–0.024 *
	Multiple	Male & Female Mice [3]	Multistage	TC05	1.4–23 mg/m^3^	Air concentration equivalence	NA	0.0048–0.079 *
OEHHA [9]	Multiple	Female MouseMale and Female Mice [3]; Male and Female Rats [7]	Multistage	NS	NS	Surface area scaling	Linear	0.077 0.002–0.16

* Linear potency estimate calculated by dividing the benchmark response rate (5%) by the TC05 value. NS = not specified; NA = not applicable; LC10 = 95% lower confidence at the concentration producing a 10% increase in risk; TC05 = total concentration associated with a 5% increase in tumor incidence.

**Table 2:** Mouse tumor data following inhalation exposure to 1,3-butadiene.

Gender	Duration (Reference)	Exposure	Concentration (ppm)	Lymphoma	Histiocytic Sarcoma	Heart	Alveolar–Bronchiolar	Forestomach	Mammary Gland	Liver	Harderian	Preputial, Ovary
Male	Acute [14]	2 h (1×)	0	7/59	NR	NR	8/59	0/59	0/59	17/59	NR	NR
			1000	8/58	NR	NR	9/58	1/58	0/58	21/58	NR	NR
			5000	8/58	NR	NR	12/57	1/58	0/58	21/58	NR	NR
			10,000	10/58	NR	NR	8/58	3/58	1/58	18/58	NR	NR
	Long-term [3]	6 h/d, 5 d/wk, 40 wk	200	8/50	5/50	15/50	36/50	3/50	NR	33/49	27/50	1/50
		6 h/d, 5 d/wk, 52 wk	312	8/50	7/50	33/50	32/50	9/50	NR	25/50	30/50	4/50
		6 h/d, 5 d/wk, 13 wk	625	22/50	2/50	7/50	28/50	7/50	NR	24/49	23/50	5/50
		6 h/d, 5 d/wk, 26 wk	625	33/50	2/50	13/50	17/50	10/50	NR	13/50	13/50	3/50
	Lifetime [3]	6 h/d, 5 d/wk, 103 wk	0	4/50	0/50	0/50	21/50	1/50	NR	21/50	6/50	0/50
			6.25	2/50	0/50	0/49	23/50	0/50	NR	23/50	7/50	0/50
			20	4/50	4/50	1/50	19/50	0/50	NR	30/50	9/50	0/50
			62.5	6/50	5/50	5/48	31/49	1/50	NR	25/48	20/50	0/50
			200	2/50	7/50	20/48	35/50	8/50	NR	33/48	31/50	5/50
			625	51/73	4/73 ^+^	4/73 ^+^	3/73 ^+^	4/73 ^+^	NR	5/72 ^+^	6/73 ^+^	0/73 ^+^
Female	Acute [14]	2 h (1×)	0	13/57	NR	NR	3/56	0/57	2/57	5/56	NR	0/53
			1000	19/56	NR	NR	4/56	1/56	1/56	6/55	NR	0/52
			5000	18/57	NR	NR	0/57	0/57	3/57	8/57	NR	1/53
			10,000	13/58	NR	NR	3/58	0/58	4/58	3/58	NR	0/56
	Lifetime [3]	6 h/d, 5 d/wk, 103 wk	0	6/50	3/50	0/50	4/50	0/50	0/50	15/49	8/50	1/49
			6.25	12/50	2/50	0/50	15/50	0/50	2/50	14/49	10/50	0/49
			20	11/50	7/50	0/50	19/50	3/50	4/50	15/50	7/50	1/48
			62.5	7/50	4/50	1/49	24/50	2/50	12/50	19/50	15/50	9/50
			200	9/50	7/50	21/50	25/50	4/50	15/50	16/50	20/50	8/50
			625	32/80	4/80 ^+^	23/80 ^+^	22/78 ^+^	22/80 ^+^	16/80 ^+^	2/80 ^+^	9/80 ^+^	6/79 ^+^

+ Due to early deaths primarily attributed to lymphomas, this dose group was excluded from dose–response modeling for other tumor types. NR = not reported.

**Table 3:** Rat tumor data following inhalation exposure to 1,3-butadiene [4,5].

				Target Tissues					
Gender	Duration	Exposure	Concentration (ppm)	Pancreas	Zymbal	Mammary	Thyroid	Glial Cell	Testis, Uterus
Male	Lifetime	6 h/d, 5 d/wk, 103 wk	0	3/100	1/100	1/100	3/100	1/100	0/100
			1000	1/100	1/100	2/100	5/100	4/100	3/100
			8000	11/100	2/100	0/100	1/100	5/100	8/100
Female	Lifetime	6 h/d, 5 d/wk, 103 wk	0	2/100	0/100	50/100	0/100	NR	1/100
			1000	0/100	0/100	79/100	4/100	NR	4/100
			8000	0/100	4/100	81/100	11/100	NR	5/100

**Table 4:** Use of hemoglobin adduct data in mice, rats, and humans to quantify species differences in internal dose of BD epoxide metabolites (adapted from Motwani and Tornqvist [10]).

	Metabolite-Specific Unit Internal Dose (nM*h per ppm*h BD) ^1^				
	Mouse		Rat		Human ^1^
Metabolite	Female	Male	Female	Male	Male
EB	13 ± 2	15 ± 2	0.77 ± 0.1	0.72 ± 0.1	0.11 ± 0.076
DEB	27 ± 7	38 ± 8	1.45 ± 0.2	1.37 ± 0.3	0.024 ± 0.020
EBD	266 ± 71	210 ± 30	19 ± 0.9	19 ± 2	52 ± 36

**^1^** Calculated as the pooled arithmetic mean ± SD using two datasets for exposed male workers (Motwani and Tornqvist [10]).

**Table 5:** Relative genotoxic potencies of BD metabolites in mammalian cells ^1^.

	Metabolite				
Endpoint	EB	DEB	EDB	In Vitro Cell System	Reference
DNA Damage	1.00	11.21	0.961	Human hepatocytes, pH 11.9	[43,44]
	1.00	4.22	0.955	Human hepatocytes, pH 9	
DNA Damage Mean ± SD	1.00	7.72 ± 4.94	0.96 ± 0.004		
Mutations	1.00	81.66	2.10	Human TK6 (HPRT)	[45]
	1.00	277.12	4.46	Human TK6 (TK)	
	1.00	58.10	0.45	Human TK6 (HPRT)	[46]
	1.00	114.83	0.71	Human TK6 (TK)	
	1.00	49.08	0.35	BB Mouse Fibroblasts	[47]
	— ^2^	— ^2^	— ^2^	BB Rat Fibroblasts	
	1.00	4.20	3.87	SA T100	[48]
Mutations Mean ± SD	1.00	97.5 ± 95.3	1.99 ± 1.81		
Micronuclei	1.00	128.28	0.58	BB Mouse Fibroblasts	[47]
	1.00	124.08	0.74	BB Rat Fibroblasts	
	— ^2^	— ^2^	— ^2^	Rat spermatids	[49]
Micronuclei Mean ± SD	1.00	126.18 ± 2.97	0.66 ± 0.12		
Overall Mean ± SD ^3^	1.00	85.28 ± 82.81	1.52 ± 1.48		

^1^ Calculated based on the ratio of linear slopes for each metabolite relative to the slope for EB assessed in the same cell test system (see Supplementary Materials). ^2^ Only DEB yielded a positive response, therefore, relative potencies were not estimated for this dataset. ^3^ Values used to support the calculation of data-derived extrapolation factors (Table 6).

**Table 6:** Data-derived extrapolation factors to quantify species differences in BD toxicokinetics (EF_AK_).

		Individual Metabolites			
Parameter (units)	Species/Extrapolation	EB	DEB	EBD	Metabolites Combined ^3^
Genotoxicity Index (nM*h per ppm*h BD) ^1^	Female Mouse	13.0	2303	404	2719
	Male Mouse	15.0	3241	319	3574
	Female Rat	0.77	124	28.8	153
	Male Rat	0.72	117	28.5	146
	Human	0.109	2.04	79.2	81.4
EF_AK_ (Unitless) ^2^	Human: Female Mouse	0.00842	0.000886	0.196	0.0300 ^4^
	Human: Male Mouse	0.00730	0.000630	0.249	0.0228 ^4^
	Human: Female Rat	0.142	0.0165	2.75	0.531 ^4^
	Human: Male Rat	0.152	0.0175	2.75	0.556 ^4^

^1^ Calculated as the product of unit internal dose value (Table 4) and relative cytotoxic potency (Table 5), units of nM*h per ppm*h BD. ^2^ Calculated as the ratio of genotoxicity indices for each species, unitless. ^3^ Calculated as the sum across metabolites. ^4^ Values used to calculate human equivalent concentrations for tumor PODs attributed to all three epoxide metabolites combined.

**Table 7:** Unit risk values based on tumors in mice and rats.

Dataset			Range of Model Fit Statistics for Individual Tumor Types		Unit Risk for Combined Tumor Types (ppm^−1^ HEC) *
Dataset	N	Range of Observation, (HEC, ppm Continuous)	*p*-Values	AICs	
Female Mouse (Table 2)	558	52–27,800	0.103–0.867	81.6–349.1	8.8 × 10^−4^ (5.7 × 10^−4^–1.2 × 10^−3^)
Male Mouse (Table 2)	756	49–36,550	0.052–0.966	35.6–337.3	3.5 × 10^−4^ (2.8 × 10^−4^–4.3 × 10^−4^)
Female Rat (Table 3)	300	336–2690	0.00016–0.969	35.7–357	6.7 × 10^−5^ (4.2 × 10^−5^–9.6 × 10^−5^)
Male Rat (Table 3)	300	321–2570	0.131–0.163	88.7–109	1.4 × 10^−5^ (7.5 × 10^−6^–2.1 × 10^−5^)

* HEC = Interspecies adjustments made assuming all three genotoxic epoxide metabolites contribute to the observed tumorigenic response in rodents.

## Data Availability

Not applicable.

## Supplementary Materials

The following are available online at https://www.mdpi.com/article/10.3390/toxics10070394/s1, Supporting Analyses for Deriving Unit Risk Values for BD.

## References

[1] Sathiakumar N., Bolaji E.B., Brill I., Chen L., Tipre M., Leader M., Arora T., Delzell E. (2021). 1,3-Butadiene, styrene and lymphohaematopoietic cancers among North American synthetic rubber polymer workers: Exposure–response analyses. Occup. Environ. Med..

[2] Sathiakumar N., Bolaji B., Brill I., Chen L., Tipre M., Leader M., Arora T., Delzell E. (2021). 1,3-Butadiene, styrene and selected outcomes among synthetic rubber polymer workers: Updated exposure-response analyses. Chem. Interact..

[3] NTP (1993). NTP Toxicology and Carcinogenesis Studies of 1,3-Butadiene (CAS No. 106-99-0) in B6C3F1 Mice (Inhalation Studies). Natl. Toxicol. Program Tech. Rep. Ser..

[4] Owen P.E., Glaister J.R., Gaunt I.F., Pullinger D.H. (1987). Inhalation Toxicity Studies With 1,3-Butadiene 3 Two Year Toxicity/Carcinogenicity Study in Rats. Am. Ind. Hyg. Assoc. J..

[5] Melnick R.L., Huff J.E. (1993). 1,3-Butadiene induces cancer in experimental animals at all concentrations from 6.25 to 8000 parts per million. IARC Sci. Publ..

[6] (2002). A Review of the Reference Dose and Reference Concentration Process.

[7] Hazleton (1981). 1,3-Butadiene: Inhalation teratogenicity study in the rat. Hazleton Laboratories Europe. Report 2788-522/3..

[8] Health Canada (2000). Priority Substances List Assessment Report.

[9] OEHHA (2013). 1,3-Butadiene Reference Exposure Levels.

[10] Motwani H.V., Törnqvist M. (2014). In vivo doses of butadiene epoxides as estimated from in vitro enzyme kinetics by using cob(I)alamin and measured hemoglobin adducts: An inter-species extrapolation approach. Toxicol. Appl. Pharmacol..

[11] (2014). Guidance for Applying Quantitative Data to Develop Data-Derived Extrapolation Factors for Interspecies and Intraspecies Extrapolation.

[12] Fred C., Tornqvist M., Granath F. (2008). Evaluation of Cancer Tests of 1,3-Butadiene Using Internal Dose, Genotoxic Potency, and a Multiplicative Risk Model. Cancer Res..

[13] (2005). Guidelines for Carcinogen Risk Assessment.

[14] Bucher J.R., Melnick R.L., Hildebrandt P.K. (1993). Lack of Carcinogenicity in Mice Exposed Once to High Concentrations of 1,3-Butadiene. J. Nat. Cancer Inst..

[15] (2020). Final Scope of the Risk Evaluation for 1,3-Butadiene.

[16] Himmelstein M.W., Acquavella J.F., Recio L., Medinsky M.A., Bond J.A. (1997). Toxicology and Epidemiology of 1,3-Butadiene. Crit. Rev. Toxicol..

[17] Albertini R.J., Srám R.J., Vacek P.M., Lynch J., Nicklas J.A., van Sittert N.J., Boogaard P.J., Henderson R.F., Swenberg J.A., Tates A.D. (2003). Biomarkers in Czech workers exposed to 1,3-butadiene: A transitional epidemiologic study. Res. Rep. Health Eff. Inst..

[18] Kirman C.R., Albertini R.J., Sweeney L.M., Gargas M.L. (2010). 1,3-Butadiene: I. Review of metabolism and the implications to human health risk assessment. Crit. Rev. Toxicol..

[19] Filser J.G., Bhowmik S., Faller T.H., Hutzler C., Kessler W., Midpanon S., Pütz C., Schuster A., Semder B., Veereshwarayya V. (2010). Quantitative Investigation on the Metabolism of 1,3-Butadiene and of Its Oxidized Metabolites in Once-through Perfused Livers of Mice and Rats. Toxicol. Sci..

[20] Csanády G.A., Guengerich F., Bond J.A. (1992). Comparison of the biotransformation of 1,3-butadiene and its metabolite, butadiene monoepoxide, by hepatic and pulmonary tissues from humans, rats and mice. Carcinogenesis.

[21] Schmidt U., Loeser E. (1985). Species differences in the formation of butadiene monoxide from 1,3-butadiene. Arch. Toxicol..

[22] Krause R.J., Elfarra A.A. (1997). Oxidation of Butadiene Monoxide tomeso-and (±)-Diepoxybutane by cDNA-Expressed Human Cytochrome P450s and by Mouse, Rat, and Human Liver Microsomes: Evidence for Preferential Hydration ofmeso-Diepoxybutane in Rat and Human Liver Microsomes. Arch. Biochem. Biophys..

[23] Bond A.J., Csanády A.G., Leavens T., Medinsky A.M. (1993). Research strategy for assessing target tissue dosimetry of 1,3-butadiene in laboratory animals and humans. IARC Sci. Publ..

[24] Kreuzer P.E., Kessler W., Welter H.F., Baur C., Filser J.G. (1991). Enzyme specific kinetics of 1,2-epoxybutene-3 in microsomes and cytosol from livers of mouse, rat, and man. Arch. Toxicol..

[25] Seaton M., Follansbee M., Bond J. (1995). Oxidation of 1,2-epoxy-3-butene to 1,2:3,4-diepoxybutane by cDNA-expressed human cytochromes P450 2E1 and 3A4 and human, mouse and rat liver microsomes. Carcinogenesis.

[26] Filser J.G., Faller T.H., Bhowmik S., Schuster A., Kessler W., Pütz C., Csanády G.A. (2001). First-pass metabolism of 1,3-butadiene in once-through perfused livers of rats and mice. Chem. Interact..

[27] Filser J.G., Hutzler C., Meischner V., Veereshwarayya V., Csanády G.A. (2007). Metabolism of 1,3-butadiene to toxicologically relevant metabolites in single-exposed mice and rats. Chem. Interact..

[28] Thornton-Manning J.R., Dahl A.R., Bechtold W.E., Griffith W.C., Pei L., Henderson R.F. (1995). Gender differences in the metabolism of 1, 3-butadiene in Sprague-Dawley rats following a low level inhalation exposure. Carcinogenesis.

[29] Thornton-Manning J.R., Dahl A.R., Bechtold W.E., Griffith W.C., Henderson R.F., Hederson R.F. (1995). Disposition of butadiene monoepoxide and butadiene diepoxide in various tissues of rats and mice following a low-level inhalation exposure to 1,3-butadiene. Carcinogenesis.

[30] Boysen G., Georgieva N.I., Upton P.B., Jayaraj K., Li Y., Walker V.E., Swenberg J.A. (2004). Analysis of Diepoxide-Specific Cyclic *N*-Terminal Globin Adducts in Mice and Rats after Inhalation Exposure to 1,3-Butadiene. Cancer Res..

[31] Swenberg J.A., Boysen G., Georgieva N., Bird M.G., Lewis R.J. (2007). Future directions in butadiene risk assessment and the role of cross-species internal dosimetry. Chem. Interact..

[32] Georgieva N.I., Boysen G., Bordeerat N., Walker V.E., Swenberg J.A. (2010). Exposure-Response of 1,2:3,4-Diepoxybutane–Specific N-Terminal Valine Adducts in Mice and Rats after Inhalation Exposure to 1,3-Butadiene. Toxicol. Sci..

[33] Albertini R.J., Sram R.J., Vacek P.M., Lynch J., Rossner P., Nicklas J.A., McDonald J.D., Boysen G., Georgieva N., Swenberg J.A. (2007). Molecular epidemiological studies in 1,3-butadiene exposed Czech workers: Female–male comparisons. Chem. Interact..

[34] Swenberg J.A., Bordeerat N.K., Boysen G., Carro S., Georgieva N.I., Nakamura J., Troutman J.M., Upton P.B., Albertini R.J., Vacek P.M. (2011). 1,3-Butadiene: Biomarkers and application to risk assessment. Chem. Interact..

[35] Boysen G., Georgieva N.I., Bordeerat N.K., Šram R.J., Vacek P., Albertini R.J., Swenberg J.A. (2011). Formation of 1,2:3,4-Diepoxybutane-Specific Hemoglobin Adducts in 1,3-Butadiene Exposed Workers. Toxicol. Sci..

[36] Kotapati S., Esades A., Matter B., Le C., Tretyakova N. (2015). High throughput HPLC–ESI−MS/MS methodology for mercapturic acid metabolites of 1,3-butadiene: Biomarkers of exposure and bioactivation. Chem. Interact..

[37] Albertini R.J., Carson M.L., Kirman C.R., Gargas M.L. (2010). 1,3-Butadiene: II. Genotoxicity profile. Crit. Rev. Toxicol..

[38] Kirman C.R., Albertini R.A., Gargas M.L. (2010). 1,3-Butadiene: III. Assessing carcinogenic modes of action. Crit. Rev. Toxicol..

[39] Walker V.E., Degner A., Carter E.W., Nicklas J.A., Walker D.M., Tretyakova N., Albertini R.J. (2019). 1,3-Butadiene metabolite 1,2,3,4 diepoxybutane induces DNA adducts and micronuclei but not t(9;22) translocations in human cells. Chem. Interact..

[40] Preston R.J. (1999). Chromosomal changes. IARC Sci. Publ..

[41] ten Berge W.F., Zwart A., Appelman L.M. (1986). Concentration—Time mortality response relationship of irritant and systemically acting vapours and gases. J. Haz. Mat..

[42] (2012). Benchmark Dose Technical Guidance.

[43] Wen Y., Zhang P.P., An J., Yu Y.X., Wu M.H., Sheng G.Y., Fu J.M., Zhang X.Y. (2011). Diepoxybutane induces the formation of DNA-DNA rather than DNA-protein cross-links, and single-strand breaks and alkali-labile sites in human hepatocyte L02 cells. Mutat. Res..

[44] Zhang P.P., Wen Y., An J., Yu Y.X., Wu M.H., Zhang X.Y. (2012). DNA damage induced by three major metabolites of 1,3-butadiene in human hepatocyte L02 cells. Mutat. Res..

[45] Meng R.Q., Hackfeld L.C., Hedge R.P., Wisse A.L., Redetzke D.L., Walker E.V. (2010). Mutagenicity of stereochemical configurations of 1,3-butadiene epoxy metabolites in human cells. Res. Rep. Health Eff. Inst..

[46] Cochrane E.J., Skopek T.R. (1993). Mutagenicity of 1,3-butadiene and its epoxide metabolites in human TK6 cells and in splenic T cells isolated from exposed B6C3F1 mice. IARC Sci. Publ..

[47] Erexson G.L., Tindall K.R. (2000). Micronuclei and gene mutations in transgenic Big Blue^®^ mouse and rat fibroblasts after exposure to the epoxide metabolites of 1,3-butadiene. Mutat. Res. Toxicol. Environ. Mutagen..

[48] Adler I.D., Kliesch U., Nylund L., Peltonen K. (1997). In vitro and in vivo mutagenicity of the butadiene metabolites butadiene diolepoxide, butadiene monoepoxide and diepoxybutane. Mutagenesis.

[49] Sjöblom T., Lähdetie J. (1996). Micronuclei are induced in rat spermatids in vitro by 1,2,3,4-diepoxybutane but not by 1,2-epoxy-3-butene and 1,2-dihydroxy-3,4-epoxybutane. Mutagenesis.

[50] Valdez-Flores C., Erraguntla N., Budinsky R.A., Cagen S., Kirman C.R. (2022). An Updated Lymphohematopoietic and Bladder Cancers Risk Evaluation for Occupational and Environmental Exposures to 1,3-Butadiene. Chem. -Biol. Interact..

[51] Park S.L., Kotapati S., Wilkens L.R., Tiirikainen M., Murphy S.E., Tretyakova N., Le Marchand L. (2014). 1,3-Butadiene Exposure and Metabolism among Japanese American, Native Hawaiian, and White Smokers. Cancer Epidemiol. Biomark. Prev..

[52] Boldry E.J., Patel Y.M., Kotapati S., Esades A., Park S.L., Tiirikainen M., Stram D.O., Le Marchand L., Tretyakova N. (2017). Genetic Determinants of 1,3-Butadiene Metabolism and Detoxification in Three Populations of Smokers with Different Risks of Lung Cancer. Cancer Epidemiol. Biomark. Prev..

[53] Sangaraju D., Boldry E.J., Patel Y.M., Walker V., Stepanov I., Stram D., Hatsukami D., Tretyakova N. (2017). Isotope Dilution nanoLC/ESI^+^-HRMS^3^ Quantitation of Urinary N7-(1-Hydroxy-3-buten-2-yl) Guanine Adducts in Humans and Their Use as Biomarkers of Exposure to 1,3-Butadiene. Chem. Res. Toxicol..

[54] Jokipii Krueger C.C., Park S.L., Madugundu G., Patel Y., Le Marchand L., Stram D.O., Tretyakova N. (2021). Ethnic differences in excretion of butadiene–DNA adducts by current smokers. Carcinogenesis.

[55] Fernandez-Salguero P., Hoffman S.M., Cholerton S., Mohrenweiser H., Raunio H., Rautio A., Pelkonen O., Huang J.D., Evans E.W., Idle J.R. (1995). A genetic polymorphism in coumarin 7-hydroxylation: Sequence of the human CYP2A genes and identification of variant CYP2A6 alleles. Am. J. Hum. Genet..

[56] Wormhoudt L.W., Commandeur J.N.M., Vermeulen N.P.E. (1999). Genetic Polymorphisms of Human*N*-Acetyltransferase, Cytochrome P450, Glutathione-S-Transferase, and Epoxide Hydrolase Enzymes: Relevance to Xenobiotic Metabolism and Toxicity. Crit. Rev. Toxicol..

[57] London S. (2000). Lung cancer risk in relation to genetic polymorphisms of microsomal epoxide hydrolase among African-Americans and Caucasians in Los Angeles County. Lung Cancer.

[58] Yoshikawa M., Hiyama K., Ishioka S., Maeda H., Maeda A., Yamakido M. (2000). Microsomal epoxide hydrolase genotypes and chronic obstructive pulmonary disease in Japanese. Int. J. Mol. Med..

[59] Degner A., Arora R., Erber L., Chao C., Peterson L.A., Tretyakova N.Y. (2020). Interindividual Differences in DNA Adduct Formation and Detoxification of 1,3-Butadiene-Derived Epoxide in Human HapMap Cell Lines. Chem. Res. Toxicol..

[60] Fustinoni S., Soleo L., Warholm M., Begemann P., Rannug A., Neumann H.-G., Swenberg A.J., Vimercati L., Colombi A. (2002). Influence of metabolic genotypes on biomarkers of exposure to 1,3-butadiene in humans. Cancer Epidemiol. Biomark. Prev..

[61] Auerbach A.D. (2015). Diagnosis of Fanconi Anemia by Diepoxybutane Analysis. Curr. Protoc. Hum. Genet..

[62] Hines R.N. (2007). Ontogeny of human hepatic cytochromes P450. J. Biochem. Mol. Toxicol..

[63] Johnsrud E.K., Koukouritaki S.B., Divakaran K., Brunengraber L.L., Hines R.N., McCarver D.G. (2003). Human hepatic CYP2E1 expression during development. J. Pharmacol. Exp. Ther..

[64] Matlock M.K., Tambe A., Elliott-Higgins J., Hines R.N., Miller G.P., Swamidass S.J. (2019). A Time-Embedding Network Models the Ontogeny of 23 Hepatic Drug Metabolizing Enzymes. Chem. Res. Toxicol..

[65] Nieto A., Zhang L., Bhandari D., Zhu W., Blount B.C., De Jesús V.R. (2021). Exposure to 1,3-Butadiene in the U.S. Population: National Health and Nutrition Examination Survey 2011–2016. Biomarkers.

[66] Kirman C.R., North C.M., Tretyakova N.Y., Erraguntla N., Shen H., Hays S.H. (2022). Use of Biomarker Data and Metabolite Relative Potencies to Support Derivation of Noncancer Reference Values for 1,3-Butadiene. Regul. Toxicol. Pharmacol..

[67] U.S. EPA (2011). Exposure Factors Handbook 2011 Edition (Final Report).

[68] IRIS Integrated Risk Information System|U.S. Environmental Protection Agency (2010). Record for Acrylamide.

[69] (2010). Development of a Relative Potency Factor (RPF) Approach for Polycyclic Aromatic Hydrocarbon (PAH) Mixtures.

[70] (2010). Recommended Toxicity Equivalence Factors (TEFs) for Human Health Risk Assessments of 2,3,7,8- Tetrachlorodibenzo-p-dioxin and Dioxin-Like Compounds.

[71] Nakamura J., Carro S., Gold A., Zhang Z. (2020). An unexpected butadiene diolepoxide-mediated genotoxicity implies alternative mechanism for 1,3-butadiene carcinogenicity. Chemosphere.

[72] Elfarra A.A., Zhang X.-Y. (2012). Alcohol Dehydrogenase- and Rat Liver Cytosol-Dependent Bioactivation of 1-Chloro-2-hydroxy-3-butene to 1-Chloro-3-buten-2-one, a Bifunctional Alkylating Agent. Chem. Res. Toxicol..

[73] Wang Y., Yu Y.-X., Luan Y., An J., Yin D.-G., Zhang X.-Y. (2018). Bioactivation of 1-chloro-2-hydroxy-3-butene, an in vitro metabolite of 1,3-butadiene, by rat liver microsomes. Chem. Interact..

[74] Wu W.-J., Tang W.-F., Xiang M.-H., Yan J., Cao X., Zhou C.-H., Chang Y., Xi J., Cao Y.-Y., Luan Y. (2019). Isotope Dilution LC/ESI(-)-MS-MS Quantitation of Urinary 1,4-Bis(N-Acetyl-S-Cysteinyl)-2-Butanone in Mice and Rats as the Biomarker Of. Chem. Interact..

