# Supplementary material for: Use of Biomarker Data and Relative Potencies of Mutagenic Metabolites to Support Derivation of Cancer Unit Risk Values for 1,3-Butadiene from Rodent Tumor Data
Source: Toxics. 2022 Jul 15;10(7):394. doi: 10.3390/toxics10070394 (PMC9316621; doi:10.3390/toxics10070394)
Supplement: Supplementary file 1 [file toxics-10-00394-s001.zip › toxics-1781524-supplementary.pdf]

toxics

MDPI

Supplementary Materials: Use of Biomarker Data and Relative Potencies of Mutagenic Metabolites to Support Derivation of Cancer Unit Risk Values for 1,3-Butadiene from Rodent Tumor Data

Christopher R Kirman and Sean M Hays

Supporting Analyses for Deriving Unit Risk Values for BD

1. Calculation of Relative Genotoxic Potencies for BD Metabolites

Data from the published literature that characterize the dose–response relationship for the genotoxicity of three reactive 1,3-butadiene (BD) metabolites (epoxybutene or EB, diepoxybutane or DEB, epoxybutane diol or EBD) in in vitro cell systems are summarized below. All digitization of data contained in figures was performed using DigitizeIt (Bormann, version 2.4.1; Braunschweig, Germany).

1.1. Datasets

Cochrane and Skopec [46]

In this study, TK6 human lymphoblastoid cells were exposed for 24 hours to 0–400 μM 1,2-epoxybutene (EB), 0–800 μM 3,4-epoxy-1,2-butanediol (EBD), or 0–6 μM 1,2,3,4-diepoxybutane (DEB) to assess their mutagenicity and cytotoxicity. Data mutagenicity (HPRT and TK loci) were digitized from Figures 5 and 6 of the paper (Table S1). $\times 10^0$

Table S1. Cytotoxicity of BD metabolites in TK6 cells [46].

		Mutation Frequency	
**Metabolite**	**μM**	**HPRT**	**TK**
DEB	0.0×10^0	0.0×10^0	0.0×10^0
	1.3×10^0	2.7×10^0	8.3×10^0
	2.6×10^0	3.7×10^0	1.8×10^1
	3.9×10^0	8.5×10^0	2.1×10^1
	5.2×10^0	9.1×10^0	3.0×10^1
EB	0.0×10^0	0.0×10^0	0.0×10^0
	$1.3x10^2$	2.8×10^0	6.1×10^0
	2.5×10^2	9.4×10^0	1.6×10^1
	3.8×10^2	$1.2x10^1$	1.7×10^1
	5.0×10^2	1.5×10^1	1.4×10^1
EBD	0.0×10^0	0.0×10^0	0.0×10^0
	1.8×10^2	1.4×10^0	2.3×10^0
	4.5×10^2	4.8×10^0	1.8×10^1
	8.1×10^2	1.1×10^1	2.6×10^1

Erexson and Tindall [47]

The goal of this study was to evaluate possible differences in the production of cytotoxicity and genetic damage in transgenic Big Blue mouse and rat fibroblasts exposed to three predominant epoxide metabolites of BD. Analyses of genetic damage (micronuclei and mutations) was assessed after in vitro exposure of cells to 125–500 μM EB, 2.5–10 μM

DEB, or 250–1000 μM EBD. Data on micronuclei and mutation frequencies were obtained from Table 1 of Erexson and Tindall [47], and presented in Table S2.

Table S2. Cytotoxicity of BD metabolites in Big Blue rat and mouse fibroblasts [47].

Metabolite	Mouse Micronuclei (%)		Mouse Mutation Frequency ($\times 10^{-5}$)		Mouse Micronuclei (%)		Mouse Mutation Frequency ($\times 10^{-5}$)	
**DEB (μM)**	**Mean**	**SD**	**Mean**	**SD**	**Mean**	**SD**	**Mean**	**SD**
0	3.00×10^{0}	8.20×10^{-1}	1.25×10^{1}	2.33×10^{0}	1.67×10^{0}	4.90×10^{-1}	7.48×10^{0}	2.70×10^{-1}
2.5	7.13×10^{0}	1.61×10^{0}	1.87×10^{1}	4.39×10^{0}	6.10×10^{0}	1.25×10^{0}	1.78×10^{1}	2.69×10^{0}
5	9.87×10^{0}	1.75×10^{0}	1.84×10^{1}	2.87×10^{0}	1.04×10^{1}	1.76×10^{0}	1.73×10^{1}	4.24×10^{0}
10	1.50×10^{1}	1.00×10^{0}	2.51×10^{1}	4.61×10^{0}	2.08×10^{1}	3.78×10^{0}	2.07×10^{1}	4.24×10^{0}
**EB (μM)**	**Mean**	**SD**	**Mean**	**SD**	**Mean**	**SD**	**Mean**	**SD**
125	4.03×10^{0}	4.90×10^{-1}	1.81×10^{1}	4.11×10^{0}	4.37×10^{0}	3.80×10^{-1}	4.81×10^{0}	7.40×10^{-1}
250	6.00×10^{0}	3.50×10^{-1}	1.82×10^{1}	5.08×10^{0}	5.77×10^{0}	6.00×10^{-1}	6.99×10^{0}	1.63×10^{0}
500	7.46×10^{0}	1.50×10^{-1}	2.51×10^{1}	3.84×10^{0}	9.63×10^{0}	6.70×10^{-1}	6.82×10^{0}	1.21×10^{0}
**EBD (μM)**	**Mean**	**SD**	**Mean**	**SD**	**Mean**	**SD**	**Mean**	**SD**
250	4.07×10^{0}	2.50×10^{-1}	1.18×10^{1}	2.30×10^{0}	5.77×10^{0}	1.86×10^{0}	5.38×10^{0}	1.28×10^{0}
500	6.13×10^{0}	9.00×10^{-1}	1.33×10^{1}	2.62×10^{0}	9.87×10^{0}	8.10×10^{-1}	4.09×10^{0}	4.30×10^{-1}
1000	8.13×10^{0}	5.90×10^{-1}	2.03×10^{1}	1.20×10^{0}	1.33×10^{1}	1.70×10^{0}	6.36×10^{0}	2.03×10^{0}

Meng et al.[45]

The objective of this study was to evaluate the cytotoxicity and mutagenicity of stereoisomers of major BD metabolites in human cells (TK6). Mutation frequencies were measured in chemical–exposed cells vs. those in control cells at the HPRT and TK loci. TK6 cells were exposed to 0, 2, 4, or 6 μM of each form of DEB, or to 0, 200, 400, or 600 μM of each form of EB for 24 hours to determine the mutagenic efficiencies. The exposure concentrations for EBD ranged from 5 to 1000 μM. Data on mutation frequencies were obtained from Tables S3 and S4 in the supplemental material for Meng et al. [45], and are presented in Tables S3 and S4.

Table S3. Mutation frequencies (HPRT) for BD metabolite stereoisomers in TK6 cells [45] .

	MF ($\times 10^{-6}$)				SD ($\times 10^{-6}$)			
**DEB (μM)**	**Meso**	**R,R**	**S,S**		**Meso**	**R,R**	**S,S**	
0.0×10^{0}	5.00×10^{0}				2.10×10^{0}			
2.0×10^{0}	1.52×10^{1}	1.10×10^{1}	1.22×10^{1}		4.40×10^{0}	4.50×10^{0}	6.50×10^{0}	
4.0×10^{0}	1.88×10^{1}	2.46×10^{1}	2.15×10^{1}		9.80×10^{0}	1.67×10^{1}	1.33×10^{1}	
6.0×10^{0}	1.87×10^{1}	2.24×10^{1}	2.57×10^{1}		1.20×10^{1}	1.14×10^{1}	1.87×10^{1}	
**EB (μM)**	**R**	**S**			**R**	**S**		
0.0×10^{0}	4.70×10^{0}				2.70×10^{0}			
2.0×10^{2}	8.90×10^{0}	9.20×10^{0}			6.30×10^{0}	1.60×10^{0}		
4.0×10^{2}	1.87×10^{1}	2.04×10^{1}			7.30×10^{0}	5.60×10^{0}		
6.0×10^{2}	2.28×10^{1}	2.91×10^{1}			7.30×10^{0}	1.02×10^{1}		
**EBD (μM)**	**R,R**	**R,S**	**S,R**	**S,S**	**R,R**	**R,S**	**S,R**	**S,S**
0.0×10^{0}	1.13×10^{1}				4.40×10^{0}			
4.0×10^{1}		1.99×10^{1}				3.10×10^{0}		
1.2×10^{2}		1.95×10^{1}				4.30×10^{0}		
2.0×10^{2}	1.24×10^{1}	1.16×10^{1}	1.16×10^{1}	1.45×10^{1}	3.50×10^{0}	4.50×10^{0}	1.90×10^{0}	2.90×10^{0}
6.0×10^{2}	1.34×10^{1}		1.04×10^{1}	1.81×10^{1}	3.80×10^{0}		1.20×10^{0}	3.70×10^{0}
1.0×10^{3}	1.01×10^{1}		1.83×10^{1}	2.30×10^{1}	1.90×10^{0}		3.50×10^{0}	1.30×10^{0}

Table S4. Mutation frequencies (TK) for BD metabolite stereoisomers in TK6 cells [45].

	**MF (× 10^{-6})**				**SD (× 10^{-6})**			
**DEB (μM)**	**Meso**	**R,R**	**S,S**		**Meso**	**R,R**	**S,S**	
0.0×10^{0}	9.60×10^{0}				3.40×10^{0}			
2.0×10^{0}	3.13×10^{1}	2.71×10^{1}	2.91×10^{1}		1.09×10^{1}	4.30×10^{0}	1.90×10^{1}	
4.0×10^{0}	3.87×10^{1}	4.64×10^{1}	4.16×10^{1}		7.90×10^{0}	1.55×10^{1}	1.73×10^{1}	
6.0×10^{0}	4.08×10^{1}	5.66×10^{1}	6.58×10^{1}		3.53×10^{1}	3.13×10^{1}	3.11×10^{1}	
**EB (μM)**	**R**	**S**			**R**	**S**		
0.0×10^{0}	8.80×10^{0}				2.70×10^{0}			
2.0×10^{2}	1.61×10^{1}	1.35×10^{1}			6.30×10^{0}	4.70×10^{0}		
4.0×10^{2}	2.65×10^{1}	2.10×10^{1}			8.30×10^{0}	5.90×10^{0}		
6.0×10^{2}	2.35×10^{1}	2.41×10^{1}			8.60×10^{0}	8.50×10^{0}		
**EBD (μM)**	**R,R**	**R,S**	**S,R**	**S,S**	**R,R**	**R,S**	**S,R**	**S,S**
0.0×10^{0}	1.45×10^{1}				3.10×10^{0}			
4.0×10^{1}		3.01×10^{1}				4.20×10^{0}		
1.2×10^{2}		2.47×10^{1}				2.60×10^{0}		
2.0×10^{2}	1.36×10^{1}	1.08×10^{1}	1.58×10^{1}	1.29×10^{1}	4.20×10^{0}	3.80×10^{0}	5.30×10^{0}	2.10×10^{0}
6.0×10^{2}	1.55×10^{1}		1.33×10^{1}	1.53×10^{1}	4.10×10^{0}		2.10×10^{0}	2.10×10^{0}
1.0×10^{3}	1.24×10^{1}		2.26×10^{1}	2.46×10^{1}	2.90×10^{0}		1.90×10^{0}	3.20×10^{0}

Adler et al. [48]

The mutagenicity of BD metabolites was assessed with and without a metabolic activation system (rat liver S9 fraction) in *S. Typhimurium* TA100 cells. Mutation data (revertants per plate) including an S9 fraction were digitized from Figures 1–3 of Adler et al. [48], and are provided in Table S5.

Table S5. Mutations from BD metabolites in Ames assay [48].

**EB (μmol/plate)**	**Revertants per plate**
0.0×10^{0}	1.2×10^{2}
6.0×10^{-1}–	1.4×10^{2}
1.3×10^{0}	1.3×10^{2}
2.5×10^{0}	1.7×10^{2}
5.1×10^{0}	2.5×10^{2}
1.0×10^{1}	3.5×10^{2}
**DEB (μmol/plate)**	**Revertants per plate**
0.0×10^{0}	1.0×10^{2}
7.0×10^{1}–	1.5×10^{2}
1.0×10^{0}	2.1×10^{2}
1.6×10^{0}	2.6×10^{2}
2.7×10^{0}	2.3×10^{2}
5.1×10^{0}	3.3×10^{1}
**EB (μmol/plate)**	**Revertants per plate**
0.0×10^{0}	1.1×10^{2}
5.0×10^{1}–	1.9×10^{2}
1.5×10^{0}	3.0×10^{2}
3.4×10^{0}	4.6×10^{2}
6.0×10^{0}	6.8×10^{2}
1.2×10^{1}	1.1×10^{3}
2.4×10^{1}	1.5×10^{3}

4.9×10^1	2.0×10^3

Sjoblom and Kahdetie [49]

Micronuclei formation was assessed in rat spermatids exposed to EB, DEB, or EBD. Of the three metabolites, only DEB was found to produce an increase. Data for micronuclei formation were obtained from Table S1 of Sjoblom and Kahdetie [49], and are presented in Table S6.

Table S6. Micronuclei formed by BD metabolites in rat spermatids[49].

Metabolite	Concentration (µmol/L)	No MN Scored	Freq MN (/1000 SPTs)	SEM
EB	0	11	2.2	0.36
	100	14	2.73	0.88
	500	9	1.8	0.63
	1000	13	2.6	0.52
DEB	0	14	2.8	0.9
	5	32	6.4	0.83
	10	62	12.4	2.15
	20	95	19	2.28
EBD	0	10	2	0.6
	10	11	2.2	0.63
	50	7	1.5	0.34
	100	8	1.6	0.65

Zhang et al. [44; Wen et al. [43]

The ability of BD metabolites to produce DNA damage in human hepatocytes was assessed using a comet assay at different pH values. Data for EB and EBD were obtained by digitizing Figures 3 and 5 of Zhang et al. [44], and data for DEB were obtained by digitizing Figures 7 and 8 from Wen et al. [43], and are provided in **Table S7**.

Table S7. DNA damage produced by BD metabolites in human hepatocytes [43,44].

	Percent Tail DNA	
**EB (µM)**	**pH 11.9**	**pH 9**
1.5×10^2	4.9×10^0	3.0×10^0
3.3×10^2	6.4×10^0	3.6×10^0
5.9×10^2	6.9×10^0	5.0×10^0
8.6×10^2	7.8×10^0	5.2×10^0
1.0×10^3	9.5×10^0	7.3×10^0
**EBD (µM)**	**pH 11.9**	**pH 9**
1.6×10^2	5.4×10^0	5.5×10^0
3.3×10^2	5.4×10^0	6.7×10^0
5.9×10^2	6.2×10^0	7.4×10^0
8.5×10^2	8.0×10^0	8.7×10^0
1.0×10^3	9.1×10^0	9.3×10^0
**DEB (µM)**	**pH 11.9**	**pH 9**
0.0×10^0	5.8×10^0	5.3×10^0
5.0×10^1	8.7×10^0	6.7×10^0
2.0×10^2	1.9×10^1	1.2×10^1
5.0×10^2	2.8×10^1	1.4×10^1

8.0×10^{2}	2.9×10^{1}	1.0×10^{1}
1.0×10^{3}	2.6×10^{1}	9.1×10^{0}

1.2. BMD Methods for Relative Potency Estimates

All benchmark dose modeling was performed using USEPA's benchmark dose software (BMDS, version 3.2; U.S. Environmental Protection Agency, Washington DC, USA). A linear model was fit to the available datasets (Tables S1–S4) to determine the slope of the best fitting line for each dataset. These slopes were used to calculate relative potency estimates, expressed as the ratio of slope values relative to EB.

1.3. Genotoxic Potency Estimates

Genotoxic potencies (e.g., slopes) and relative potency estimates (i.e., relative to EB slope) are summarized in Table S5.

Table S8. Relative potency estimates for BD metabolites.

			Linear Slopes (BMDS; units are study–specific)			Relative Potency (unitless)		
**Endpoint**	**Reference**	**Cell line**	**EB**	**DEB**	**EBD**	**EB**	**DEB**	**EBD**
DNA Damage	[43,44]	Human hepatocyte, pH 11.9	4.01×10^{-3}–	4.49×10^{-2}–	3.85×10^{-3}–	1.00×10^{0}	1.12×10^{1}	9.61×10^{-1}–
		Human hepatocyte, pH 9	3.88×10^{-3}–	1.64×10^{-2}–	3.70×10^{-3}–	1.00×10^{0}	4.22×10^{0}	9.55×10^{-1}–
Mutations	[48]	SA T100	2.37×10^{1}	9.98×10^{1}	9.18×10^{1}	1.00×10^{0}	4.20×10^{0}	3.87×10^{0}
	[45]	Human TK6 cells, hprt	3.71×10^{-2}–	3.03×10^{0}	7.81×10^{-2}–	1.00×10^{0}	8.17×10^{1}	2.10×10^{0}
		Human TK6 cells, tk	2.70×10^{-2}–	7.48×10^{0}	1.20×10^{-1}–	1.00×10^{0}	2.77×10^{2}	4.46×10^{0}
	[46]	Human TK6 cells, hprt	3.17×10^{-2}–	1.84×10^{0}	1.43×10^{-2}–	1.00×10^{0}	5.81×10^{1}	4.50×10^{-1}–
	[46]	Human TK6 cells, tk	4.86×10^{-2}–	5.58×10^{0}	3.44×10^{-2}–	1.00×10^{0}	1.15×10^{2}	7.07×10^{-1}–
	[47]	Big Blue mouse fibroblasts	2.34×10^{-2}–	1.15×10^{0}	8.30×10^{-3}–	1.00×10^{0}	4.91×10^{1}	3.55×10^{-1}–
	[47]	Big Blue rat fibroblasts	Only DEB was positive (no EB potency for calculation)					
MN	[49]	Rat spermatids	Only DEB was positive (no EB potency for calculation)					
	[47]	Big Blue mouse fibroblasts	9.15×10^{-3}–	1.17×10^{0}	5.27×10^{-3}–	1.00×10^{0}	1.28×10^{2}	5.75×10^{-1}–
	[47]	Big Blue rat fibroblasts	1.55×10^{-2}–	1.92×10^{0}	1.15×10^{-2}–	1.00×10^{0}	1.24×10^{2}	7.41×10^{-1}–
Combined	DNA Damage		mean			1.00×10^{0}	7.72×10^{0}	9.58×10^{-1}–
	Mutations		mean			1.00×10^{0}	9.75×10^{1}	1.99×10^{0}
	MN		mean			1.00×10^{0}	1.26×10^{2}	6.58×10^{-1}–
	Overall		mean			1.00×10^{0}	8.53×10^{1}	1.52×10^{0}

2. Concentration x Time Analysis of Male Mouse Tumors

Stop–exposure study data for select tumors (heart, lung, lymphoma) in male mice exposed to BD (see Table 2 of main body of the paper) were used to assess concentration (C) x time or duration (T) relationships based on the methods of ten Berge [41], using the following steps:

1. A multistage model (BMDS, version 3.2) was fit to the dose–response data from the lifetime cancer bioassay (Table 2) to estimate a point of departure (EC10).

2. The degree of the multistage model from step 1 (**Table S9** was applied separately to each duration from the stop–exposure study (single data point). The multistage model parameters were scaled until the predicted incidence matched the observed incidence. The predicted EC10 for the duration was reported. A comparison of observed vs. predicted tumor incidences is provided in Figure S1.
3. For each tumor type, a log–log plot of the predicted EC10 values (ppm) vs. duration (days) was prepared in Microsoft Excel (version 16.56). A power regression model was fit to the EC10 values and the slope of the model (exponent) was recorded for interpretation (see text in main body of the paper)

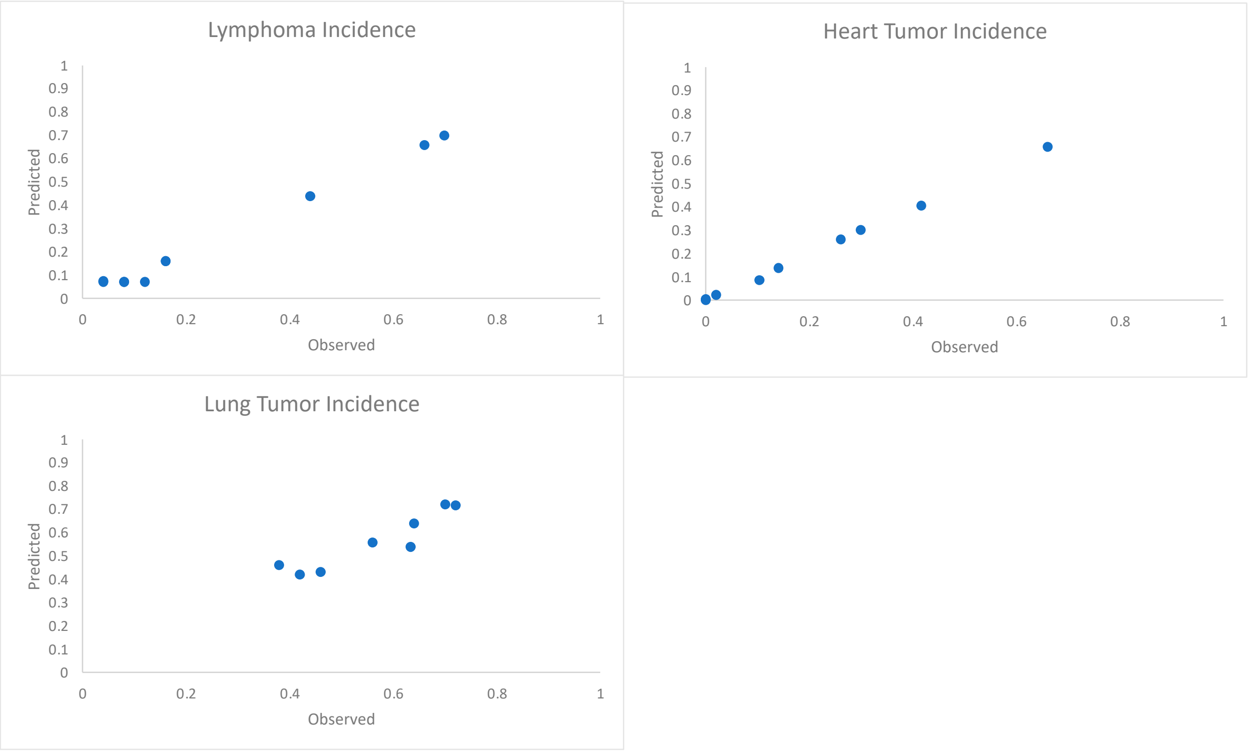

Figure S1. Observed vs. predicted (multistage model) tumor incidence values for select tumors in male mice for all exposure durations (lifetime and stop–exposure studies).

3. BMD Modeling of Individual Tumor Types

A multistage model (BMDS, version 3.2; U.S. Environmental Protection Agency, Washington DC, USA) was fit to the lifetime cancer bioassay data for each rodent tumor dataset individually (see Tables 2 and 3 of the main body of the paper). Multistage model parameters for each dataset are provided in Table S9.

Table S9. Multistage model parameters for individual tumor types in rats and mice.

		Multistage Model Term					
**Sex, Species**	**Tumor Type**	**Background**	**b1**	**b2**	**b3**	**b4**	**b5**
Male Mouse	Kidney	6.7×10^{-3}–	—	—	2.3×10^{-7}–		
	Preputial	—	—	—	—	6.5×10^{-11}–	
	Harderian	1.2×10^{-1}–	4.6×10^{-3}–				
	Liver	4.7×10^{-1}–	2.7×10^{-3}–				
	Forestomach	6.5×10^{-3}–	—	4.1×10^{-6}–			
	Lung	4.2×10^{-1}–	3.7×10^{-3}–				
	Heart	—	9.0E–04	8.5×10^{-6}–			

	Histiosarcoma	7.1×10^{-3}–	1.1×10^{-3}–				
	Lymphoma	7.2×10^{-2}–	—	—	—	—	1.2×10^{-14}–
Female Mouse	Mammary	1.6×10^{-2}–	2.4×10^{-3}–				
	Ovary	6.5×10^{-2}–	1.7×10^{-3}–				
	Harderian	1.6×10^{-1}–	1.8×10^{-3}–				
	Liver	3.1×10^{-1}–	2.0×10^{-4}–				
	Forestomach	9.0×10^{-3}–	5.0×10^{-4}–				
	Lung	1.1×10^{-1}–	2.2×10^{-2}–				
	Heart	—	—	1.2×10^{-5}–			
	Histiosarcoma	7.1×10^{-2}–	4.0×10^{-4}–				
	Lymphoma	1.8×10^{-1}–	—	—	—	—	3.3×10^{-15}–
Male Rat	Testis	2.1×10^{-3}–	1.2×10^{-5}–				
	Pancreas	2.0×10^{-2}–	—	1.3×10^{-9}–			
	Brain	2.1×10^{-2}–	4.3×10^{-6}–				
Female Rat	Uterus	1.3×10^{-2}–	5.9×10^{-6}–				
	Mammary	6.1×10^{-1}–	1.0×10^{-4}–				
	Thyroid	1.9×10^{-3}–	1.7×10^{-5}–				
	Zymbal	—	—	6.3×10^{-10}–			

The multistage model was used to estimate a distribution for the points of departure (EC10 values), and a distribution for the unit risk value for each tumor type was calculated by dividing the benchmark response rate (10%) by the point of departure. An example of the distribution of unit risk values for each tumor type is provided for female mice in Figure S2.

(A)

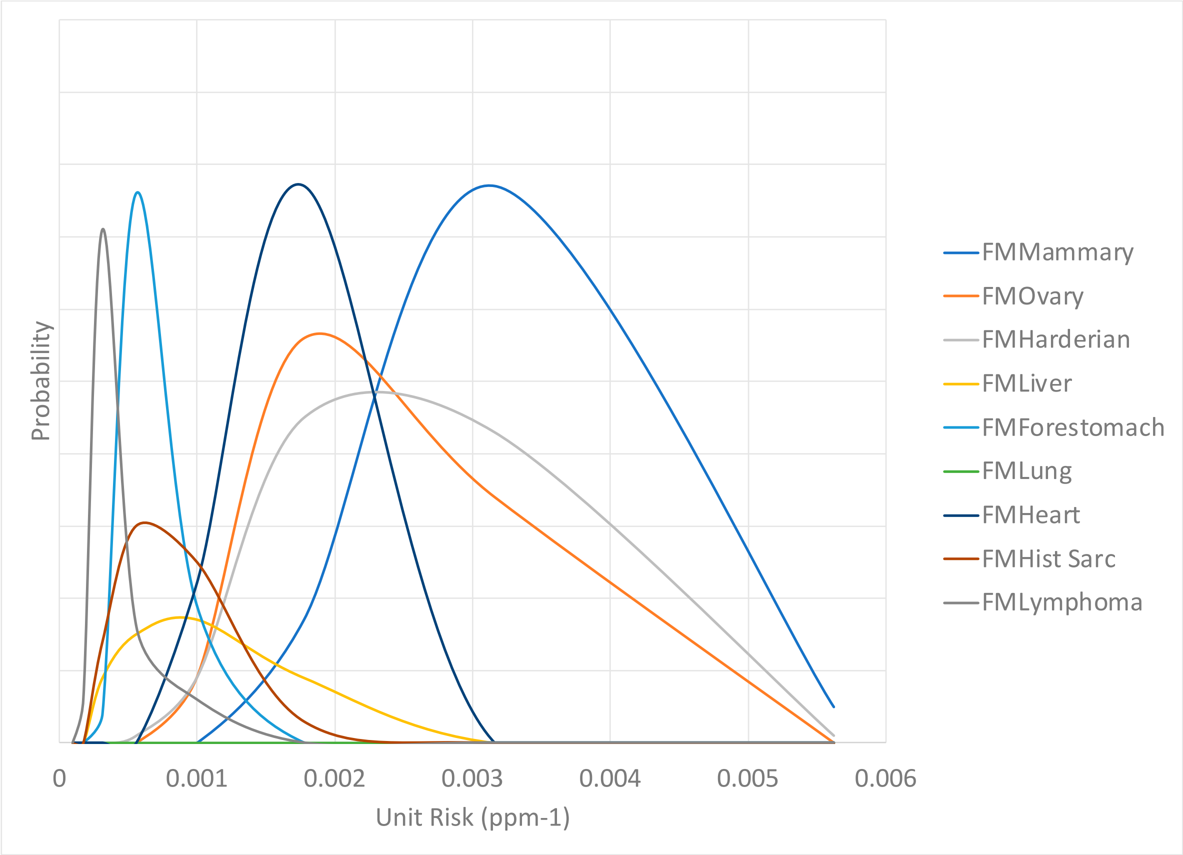

(B)

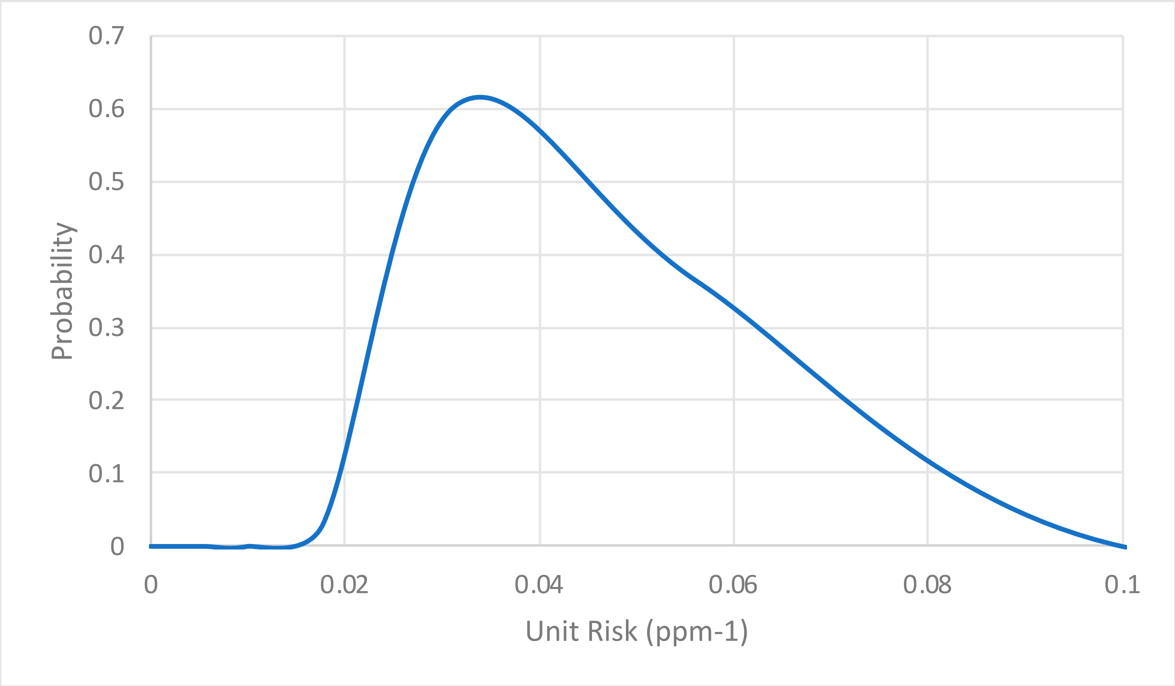

Figure S2. Distributions for unit risks based on female mice: (**A**) individual tumors; (**B**) combined tumors.

A distribution for the multisite unit risk value was calculated for each species/sex by summing across cancer endpoints:

$$UR_{Combined} = \sum(UR_{Endpoint1} + UR_{Endpoint2} \cdots)$$

where

$UR_{Combined}$ = combined unit risk across endpoint calculated for each sex and species (ppm^{-1});

$UR_{Endpoint}$ = tumor endpoint specific unit risk within each sex/species (ppm^{-1}).

A distribution for the combined UR values was generated using Monte Carlo methods (Crystal Ball; Excel; version 7.3 ; Oracle, Austin TX, USA) based on a simulation of 10,000 iterations. The 5th and 95th percentiles for the combined UR distributions were adopted as the lower and upper confidence limits, respectively, for each combined dataset. Resulting distributions are provided in Figure 4 of the main paper.
